# Polymorphisms of the *SIPA1 *gene and sporadic breast cancer susceptibility

**DOI:** 10.1186/1471-2407-9-331

**Published:** 2009-09-18

**Authors:** Szu-Min Hsieh, Robert A Smith, Nicholas A Lintell, Kent W Hunter, Lyn R Griffiths

**Affiliations:** 1 Laboratory of Cancer Biology and Genetics, National Cancer Institute, National Institutes of Health, Bethesda, MD 20892, USA; 2 Griffith Institute for Health and Medical Research, Griffith University, Gold Coast Campus, Australia

## Abstract

**Background:**

The novel breast cancer metastasis modulator gene signal-induced proliferation-associated 1 (*Sipa1*) underlies the breast cancer metastasis efficiency modifier locus Mtes 1 and has been shown to influence mammary tumour metastatic efficiency in the mouse, with an ectopically expressing *Sipa1 *cell line developing 1.5 to 2 fold more surface pulmonary metastases. *Sipa1 *encodes a mitogen-inducible GTPase activating (GAP) protein for members of the Ras-related proteins; participates in cell adhesion and modulates mitogen-induced cell cycle progression. Germline *SIPA1 *SNPs showed association with positive lymph node metastasis and hormonal receptor status in a Caucasian cohort. We hypothesized that *SIPA1 *may also be correlated to breast carcinoma incidence as well as prognosis. Therefore, this study investigated the potential relationship of *SIPA1 *and human breast cancer incidence by a germline SNP genotype frequency association study in a case-control Caucasian cohort in Queensland, Australia.

**Methods:**

The SNPs genotyped in this study were identified in a previous study and the genotyping assays were carried out using TaqMan SNP Genotyping Assays. The data were analysed with chi-square method and the Monte Carlo style CLUMP analysis program.

**Results:**

Results indicated significance with *SIPA1 *SNP rs3741378; the CC genotype was more frequently observed in the breast cancer group compared to the disease-free control group, indicating the variant C allele was associated with increased breast cancer incidence.

**Conclusion:**

This observation indicates SNP rs3741378 as a novel potential sporadic breast cancer predisposition SNP. While it showed association with hormonal receptor status in breast cancer group in a previous pilot study, this exonic missense SNP (Ser (S) to Phe (F)) changes a hydrophilic residue (S) to a hydrophobic residue (F) and may significantly alter the protein functions of *SIPA1 *in breast tumourgenesis. *SIPA1 *SNPs rs931127 (5' near gene), and rs746429 (synonymous (Ala (A) to Ala (A)), did not show significant associations with breast cancer incidence, yet were associated with lymph node metastasis in the previous study. This suggests that *SIPA1 *may be involved in different stages of breast carcinogenesis and since this study replicates a previous study of the associated SNP, it implicates variants of the *SIPA1 *gene as playing a potential role in breast cancer.

## Background

The novel breast cancer gene *SIPA1 *was originally identified as a candidate gene for breast cancer metastasis from mouse studies. Mouse *Sipa1 *was established as a candidate for underlying the breast cancer metastasis efficiency modifier locus Mtes 1 by Park et al [1]. The Mtes 1 loci in the mouse genome were recognized as a genetic region that substantially influenced the metastatic efficiency of mammary tumours in the mouse. The mouse Mtes 1 locus is orthologous to human chromosome 11q12-11q13, which is known to harbor the metastasis suppressor gene *BRMS1*. Utilizing a Multiple Cross Mapping strategy, mouse *Sipa1 *was identified as a potential candidate for the Mtes 1 locus and molecular research into *Sipa1 *revealed that cellular *Sipa1 *levels were correlated with cellular metastatic capacity. Signal-induced proliferation-associated 1 is further recognized as a metastasis modulator gene, since an ectopically expressing *Sipa1 *cell line developed about two fold more surface pulmonary metastases compared to the control cell line [1].

Signal-induced proliferation-associated 1 is a mitogen-inducible GTPase activating (GAP) protein for members of the Ras-related proteins; Rap1 and Rap2, but not for Ras, Rho, Cdc42, Rac and Ran, with comparable specific activity to the rap1GAP encoded protein [2]. It was found that the *SIPA1 *protein severely impedes mitogen-induced cell cycle progression when abnormally and/or prematurely expressed [3]. Signal-induced proliferation-associated 1 was also found to participate in cell adhesion via interaction with Rap1GTP activities. Park et al., 2005 demonstrated that *Sipa1 *suppression (via RNAi) induces increased cell adhesion [1]. This study reported that a single nucleotide polymorphism (SNP) in the mouse *Sipa1 *gene affects the binding efficiency of the protein to its partner protein. This polymorphism was identified in the PDZ binding domain of the *Sipa1 *protein between the mouse DBA strain and the mouse FVB strain, with the binding efficiency of the *Sipa1 *protein is higher in the mouse DBA strain allele compared to the mouse FVB strain allele [1]. A recent study also reported that germline SNPs of the *SIPA1 *gene are associated with major clinical characteristics, such as estrogen receptor status and lymph node metastasis in human breast cancer. The three SNPs rs931127 (Lymph Node metastasis, p = 0.0139), rs3741378 (ER status, p = 0.006; PR status, p = 0.035) and rs746429 (Lymph Node metastasis, p = 0.0074) from the *SIPA1 *gene were reported to be linked with different clinical characteristics in a cohort from Southern California, USA [4].

The *SIPA1 *protein has been related to increased breast cancer metastasis in the mouse model. Polymorphisms of the *Sipa1 *gene have significant effects on protein function in the mouse model and SNPs in the human *SIPA1 *gene are associated with major clinical markers. As a previous study showed that *SIPA1 *RNAi down-regulation increased the primary tumour burden in the mouse model, and given the previous observations that the *Sipa1 *gene and protein are associated with poor-prognosis markers and metastasis, we considered the possibility that the *SIPA1 *gene may be correlated to breast carcinoma incidence as well as prognosis. Therefore, this study investigated the potential relationship between *SIPA1 *and the incidence of human breast cancer by studying germline SNP frequency (based on the common genotype representing common disease phenotype hypothesis), utilizing a case-control cohort from a European descendent population in Queensland, Australia.

## Results

A previous human epidemiology pilot study showed that SNPs (rs931127, rs3741378 and rs746429) from the *SIPA1 *gene are associated with important clinical markers such as oestrogen receptor status and lymph node metastasis. Molecular studies also showed that *Sipa1 *protein levels in the cell are correlated to cell homeostasis and metastasis strength in the mouse model and the polymorphism in the *Sipa1 *protein binding region influence the protein binding efficiency. Increased primary breast tumour size was also reported in the previous mouse model study, which indicates that it is valuable to examine the possibility of a role for the *SIPA1 *gene in the initial stage of breast carcinoma in humans.

The three selected SNPs were genotyped in both the Breast Cancer group and the Control Group. The frequencies of the genotypes of SNP rs931127 are listed in Table 1. Chi-Square analysis of this data showed that no significant frequency difference was observed between the Breast Cancer Group and the Control Group samples (χ^2 ^= 0.73, df = 2, P = 0.695).

**Table 1:** Allele Frequencies for tested SNPs, including reported frequencies for CAUC 1 population.

			Genotype Frequency			
rs931127	**Group**	**Frequency**	**AA**	**AG**	**GG**	**Total**
	
	Breast Cancer	N	58	89	36	183
		
		%	31.69%	48.63%	19.67%	100%
	
	Control	N	52	97	34	183
		
		%	28.42%	53.01%	18.58%	100%
	
	CAUC 1	%	*30%*	*60%*	*10%*	*100%*

rs3741378	**Group**	**Frequency**	**TT**	**TC**	**CC**	**Total**
	
	Breast Cancer	N	6	41	131	162
		
		%	3.7%	25.31%	80.86%	100%
	
	Control	N	1	55	106	178
		
		%	0.56%	30.89%	59.55%	100%
	
	CAUC 1	%	*3.2%*	*22.6%*	*74.2%*	*100%*

rs746429	**Group**	**Frequency**	**GG**	**AG**	**AA**	**Total**
	
	Breast Cancer	N	73	91	19	183
		
		%	39.89%	49.73%	10.38%	100%
	
	Control	N	74	78	22	174
		
		%	42.53%	44.83%	12.64%	100%
	
	CAUC 1	%	*37.9%*	*48.3%*	*13.8%*	*100%*

The genotype frequency of SNP rs746429 was analysed; Chi-Square = 1, degrees of freedom = 2 with significance = 0.601. No significant frequency difference between the Breast Cancer group and the Control group was observed for this SNP.

The frequencies of the genotypes of SNP rs3741378 are also listed in Table 1. Significant frequency differences were observed between the Breast Cancer Group and the Control Group samples for this SNP. Chi-square analysis showed that the CC genotype of rs3741378 is more frequently observed in the Breast Cancer group compared to the disease-free Control group. Furthermore, the data shows that the TC genotype is more common in the controls than the cancers, which indicates that having both alleles may be protective in some manner. Due to the extreme low counts for the TT allele, Hardy-Weinberg equilibrium analysis was performed to exclude the possibility of experimental artefacts misleading the results. The result of this and the Chi-square analysis for rs3741378 can be found in table 2. Other analyses were non-significant at p = 0.607 and 0.695 for rs746429 and rs931127, respectively.

**Table 2:** Analysis Results, rs3741378 Chi-square and HW-equilibrium for all SNPs.

Chi-Square	Degrees of Freedom	Significance
75.1	2	0.023

**SNP**	**HW- Probability: BC Group**	**HW- Probability: Controls**

rs3741378	0.224	0.029

rs931127	0.34	0.859

*rs746429*	0.837	0.226

Additionally, because of the extremely low count for the TT genotype, the standard Chi-square method's assumptions are violated (requiring counts of at least 5 in all categories). Thus, a further analysis using CLUMP, a computer method similar to Chi-square analysis that uses a Monte-Carlo style probabilities rather than a set probability formula was performed. The results of CLUMP analysis confirm the original Chi-square results and are listed in Table 3. It should be noted that one of the additional analysis modes employed by CLUMP, which attempts to lower error by collapsing low count groups into the next lowest count did have significance above the 0.05 threshold, though only by a very small amount.

**Table 3:** CLUMP analysis of rs3741378.

Rs3741378	T1 analysis (mimics standard Chi-Square)	T4 analysis (collapses low-count categories into the next lowest category)
Chi-Squared stat	7.5139	4.9882

Degrees of freedom	2	1

***Probability***	0.0234	0.0503

## Discussion

Signal-induced proliferation-associated 1 was first identified as a potential breast cancer metastasis modulator in the mouse model. Molecular studies on this gene indicate that it plays an important role in regulating cell adhesion and modulating breast cancer metastasis. A preliminary human epidemiology study also showed that germline polymorphisms in the *SIPA1 *gene are correlated with several major clinical characteristics, such as estrogen receptor and lymph node metastasis status.

This study utilized a larger case-control population to investigate the three SNPs published previously, in relation to breast cancer incidence. Of the three SNPs tested, only rs3741378 showed a significant difference in frequency between the breast cancer group and the disease free control group. Since the CC genotype of this SNP was observed to be more frequent in the Breast Cancer group compared to the disease-free Control group and the TC heterozygous genotype was more common in controls than the cancer group, it indicates that the heterozygous genotype may be protective against breast cancer (Odds Ratio 0.5822, 95% CI 0.3614 to 0.938). This observation also indicates that SNP rs3741378 may play a role as a potential sporadic breast cancer predisposition gene/SNP. In the previous pilot study this SNP showed a significant correlation to both oestrogen receptor and progesterone receptor status. It is possible that this exonic missense SNP which has a Ser (S) to Phe (F) change, altering a hydrophilic residue (S) to a hydrophobic residue (F) significantly changes the protein functions of *SIPA1*. This may thus have a functional role in the hormonal status biology of breast carcinogenesis. The observed accumulation of CT genotypes in controls may be a reflection of increased substrate interactivity of the *SIPA1 *proteins derived from CT genotype cells, through interactions with different mitogenic pathways or alterations to the gene's cell adhesion functions. This SNP has been previously associated with oestrogen and progesterone receptor status, and it is possible that some of the protective functions of a CT genotype may be mediated through effects on these pathways.

It is important to note, however, that the low counts for the rare TT genotype in this population may be violating statistical assumptions and indicating a false relationship between this polymorphism and breast cancer risk. The non-parametric CLUMP analysis agreed with the initial Chi-square analysis, adding weight to the possibility of the observed relationship. The significant Hardy-Weinberg result for the control population throws some support to the possibility of a false relationship, perhaps driven by selection bias. This, however, may be an effect of the extreme rarity of the TT genotype, as both populations are within Hardy-Weinberg equilibrium for all other SNPs, indicating little or no selection bias for these SNPs. The T4 statistic collapses the lowest expected counts into the next category, somewhat abrogating the effect of the rarity of the TT genotype and its extreme closeness to significance does argue for some veracity to the observed relationship. The failure of the T4 statistic of CLUMP to reach the χ^2 ^significance of 0.05, even by the tiny margin observed, is an indicator that additional studies in larger, but still tightly localised populations should be carried out to more accurately determine the strength and nature of the effect that this *SIPA1 *SNP has on breast cancer risk. In addition, there was insufficiently detailed ER and PgR status, or other epidemiological information for the population to allow meaningful analysis in this present study. Future larger studies that have this information available are warranted.

Furthermore, the other two SNPs of *SIPA1 *screened; rs931127 which is a 5' near gene SNP and rs746429 which is a synonymous (Ala (A) to Ala (A)) SNP, did not show significant association with breast cancer, yet were reported to be associated with lymph node metastasis in the previous study. This suggests that *SIPA1 *may have different or shifted function in different stages of breast carcinogenesis, with different domains; having the increased tumourgenesis susceptibility function with the domain where SNP rs3741378 resides and promotes metastasis via the domain SNP rs746429 resides.

A recent case-control study from Gaudet et al. reported a similar trend, though the difference in their population did not reach statistical significance. The Gaudet et al. group explored the same SNPs as this research in a Polish (1995 cases, 2296 control) and a British cohort (2142 cases, 2257 controls). They reported a suggestion of an increased risk of breast cancer associated with the TT genotype of the SNP rs3741378 [5]. Both the Gaudet group and this research identified a potential relationship between this SNP and breast cancer risk. The inconsistency of the significance of the association between this study and the Gaudet is likely best explained by the diverse nature of the populations studied, through either environmental or genetic variance. The ratio of the TT genotype in the control samples has a wider range of diversity between the Gaudet study and this investigation, with the TT genotype comprising 0.62% of the control group for this study compared to 1.8% in the pooled study from the Gaudet group, both of which are less than the CAUC 1 population, indicating that this SNP may be highly variable in different areas. The difference in this rare allele frequency may explain the slight inconsistency in strength of association identified between the researches. However, the two independent groups did identifying a similar relationship, only differing in the strength and significance of the relationship identified. This, as well as previous functional work, adds support to the involvement of the *SIPA1 *gene and its SNPs in breast cancer susceptibility.

## Conclusion

The novel breast carcinogenesis gene *SIPA1 *has important molecular functions as a breast cancer metastasis modulator. A preliminary human pilot epidemiology study indicated that germline single nucleotide polymorphisms of *SIPA1 *are significantly correlated to major clinical factors, such as estrogen receptor status and increased lymph node metastasis. This study provides observations that one of these SNPs may also act as a breast cancer predisposition marker. Further molecular functional analysis of *SIPA1 *and its SNPs in the human population is needed to properly elaborate the function of this gene and its potential as breast carcinogenesis marker. This study highlights the importance of SNPs of the *SIPA1 *gene in breast carcinoma and that the screened SNPs are not only markers of poor prognosis as previously described, but may also act as predisposition markers. The protein expression level of *SIPA1 *in the mouse model has been linked to breast cancer metastasis propensity as increased levels lead to increased metastasis and decreased levels lead to a decreased amount of lung metastasis. This indicates that *SIPA1 *transcription at the genetic, expression and protein level may play an integral role in breast carcinoma and represent a key factor in the evolution of this disease. Additionally, this research further supported the hypothesis that inherited subtle genetic variations may be associated with not only with cancer metastasis but also with cancer predisposition.

## Methods

### Study Cohort

The population screened was comprised of 200 female individuals diagnosed with breast cancer and a control population of 200 females with no cancer history at all. The affected and control populations were matched for age and all were of Caucasian ethnicity, as has been previously described [6]. No other risk factors were controlled for. Samples were recruited through collaboration with the Pathology Department of the Gold Coast Hospital, Queensland Australia and additional affected samples, as well as the entire control population, were obtained through the Genomics Research Centre of Griffith University [6]. All participants gave informed consent. The study was conducted under the approval of the Gold Coast Hospital and Griffith University Ethics Committees. The reference numbers for these approvals are 9702 and MSC/07/08/HREC, respectively.

### Genotyping

The SNPs genotyped in this study were identified in a previous study [4], and were chosen based on the genomic location of known SNPs (from the NCBI SNP database) that are within the regulatory or coding regions of *SIPA1 *[4]. The NCBI SNP designation rs931127 polymorphism (-313G>A) is 313 base pairs upstream of the 5'-untranslated region of *SIPA1 *and considered as within the promoter region of the gene. The other two SNPs are located within coding regions, rs3741378 is a missense SNP within exon 1 (545C>T [F182S]) and rs746429 is a synonymous SNP within exon 12 (2760G>A [A920A]). Quality control for genotyping was provided by including 3 samples of CEPH family DNA (mention commercial source of DNA), repeated 4 times in each PCR plate. Genotyping results for these four samples were identical for all PCRs and concordance rates were 100%.

SNP analysis was carried out using TaqMan Universal PCR Master Mix (Applied Biosystems, USA) and TaqMan SNP Genotyping Assays (Applied Biosystems, USA).

The TaqMan SNP Genotyping Assays are composed of primer and probes that are pre-designed and validated by the company. The probes are conjugated with VIC-MGB or FAM-MGB dyes, one to each allele. Once the PCR is complete the 7900HT Sequencing Detection System (Applied Biosystem, USA) distributes the data points according to the signals generated depending on the allele composition of each patient. The genotype determination was made by a signal distribution comparison to several control samples (derived from CEPH Family DNA) for which the genotypes are known. Assays were carried out following the instructions from the company. The amount of volume required for each 5 μl reaction was as follows, (10 ng of DNA needs to be dried before adding the other reagents), TaqMan Universal PCR Master Mix; 2.5 μl, TaqMan SNP Genotyping Assay; 0.25 μl and 2.25 μl of DEPC Treated Water (Quality Biological, INC, USA). The reaction conditions were as follows; 95°C for 10 minutes, and 50 cycles of 92°C for 15 seconds and 60°C for 1 minute. After the PCR was finished, the plates were then read by the 7900HT Sequencing Detection System, according to manufacturer protocols (Applied Biosystems, USA).

### Statistical Analyses

To determine whether any significant differences in polymorphism frequencies occurred between the case and control populations, allele and genotype frequencies were compared using the chi-square method and the Monte Carlo style CLUMP analysis program [6,7]. The study was estimated to have an approximately 75% power to detect positive association of a SNP with low effect.

## Competing interests

The authors declare that they have no competing interests.

## Authors' contributions

KWH and LRG designed research; SMH and NAL performed experimental research; SMH and RAS analysed data; and all authors contributed to the final manuscript.

## Pre-publication history

The pre-publication history for this paper can be accessed here:

http://www.biomedcentral.com/1471-2407/9/331/prepub
